# Dissecting the mechanism of action of the adjuvant system AS01 in human immune cells: synergy of QS-21 and monophosphoryl lipid A and role of type I interferons

**DOI:** 10.3389/fimmu.2026.1916491

**Published:** 2026-09-24

**Authors:** Michelle Poe, Nickolas Neokosmidis, Juan F. Hernandez-Franco, Alyssa Q. Stiving, Emory M. Payne, Shailaa Kummar, Jian Xiong, Angela Wagner, David A. Foley, Daniel J. DiStefano, Hualin Helen Li, Harm HogenEsch

**Affiliations:** 1 Department of Comparative Pathobiology, College of Veterinary Medicine, Purdue University, West Lafayette, IN, United States; 2 Analytical Research and Development, Merck & Co., Inc., West Point, PA, United States; 3 Vaccine Drug Product Development, Merck & Co., Inc., West Point, PA, United States; 4 Purdue Institute of Inflammation, Immunology and Infectious Diseases, Purdue University, West Lafayette, IN, United States

**Keywords:** adjuvants, AS01, MPLA, peripheral blood mononuclear cells, QS-21, THP-1

## Abstract

The adjuvant system AS01 comprised of the TLR4 agonist monophosphoryl lipid A (MPLA) and the saponin QS-21 embedded in liposomes is currently used in recombinant vaccines against shingles, respiratory syncytial virus infection, and malaria. The efficacy of these vaccines is attributed to the strong immunostimulatory effect of AS01. While preclinical studies have shed light on the mechanisms by which AS01 enhances immune responses, few studies have been conducted with human immune cells to dissect how the two immunostimulants work individually and in combination. We investigated the effect of AS01 on human THP-1 cells and peripheral blood mononuclear cells (PBMCs) and found that it induced secretion of a broad panel of chemokines and a modest number of cytokines. Among the chemokines, CCL3 and CCL4 were found to have the greatest fold increase. Inhibition of TLR4 signaling revealed variable dependence on TLR4, while experiments with liposomes formulated with a TLR4 agonist, QS-21, or both demonstrated synergistic effects on the induction of many chemokines and TNF. AS01 induced strong expression of interferon-stimulated genes; this was greatly reduced by treatment with an antibody that blocks the type I interferon (IFN) receptor, indicating that AS01 induced a type I IFN-associated transcriptional signature that is partially dependent on signaling via the type I IFN receptor. AS01 selectively increased the expression of CD83 on THP-1 cells and this effect was found to be mostly driven by QS-21. This work provides novel insights into the mechanisms by which AS01 stimulates human immune cells.

## Introduction

The use of purified or recombinant protein antigens improves the safety and tolerability of vaccines compared to live attenuated or inactivated viral vaccines while it facilitates their formulation, production and characterization. However, such antigens are often poorly immunogenic on their own and require the addition of adjuvants, substances that enhance the immune response to vaccine antigens (1–3). Adjuvants are also used to induce a protective immune response in individuals with less responsive immune systems because of age, underlying disease or medical treatment. Furthermore, the addition of adjuvants may allow for a reduced amount of antigen in vaccines and increase the duration of protective immune responses. Adjuvants in licensed vaccines are heterogeneous in chemical and physical composition and include aluminum salts, oil-in-water emulsions, saponins and Toll-like receptor (TLR) agonists or combinations of these (4, 5). Many adjuvants were developed empirically and their mechanism of action (MoA) remains only partially understood (2, 3).

The adjuvant AS01 is used in a vaccine against malaria and in vaccines that target the elderly population, namely shingles (Varicella zoster) and respiratory syncytial virus (6). The adjuvant contains the TLR4 agonist monophosphoryl lipid A (MPLA) and the saponin QS-21, formulated into liposomes (LPs) composed of dioleoyl phosphatidylcholine and cholesterol in a phosphate buffer that are approximately 106 nm in diameter (7). MPLA is prepared by sequential acid and base hydrolysis of the lipopolysaccharide (LPS) of *Salmonella enterica* serovar Minnesota, resulting in a complex mixture of congeners with different numbers of acyl groups (8). MPLA and LPS are both ligands of TLR4, but MPLA has reduced toxicity compared with LPS (9). Activation of TLR4 by LPS engages the adaptor molecules MyD88 and TRIF resulting in the secretion of pro-inflammatory cytokines and type I interferons, respectively. Studies in mice attributed the reduced toxicity of MPLA to a decreased ability to signal via MyD88 resulting in lower production of inflammatory cytokines without affecting the ability to enhance the adaptive immune response (10). However, there are marked species-specific differences in TLR4 signaling as certain MPLA congeners are inhibitory for human TLR4, but not mouse TLR4, resulting in an overall lower response of human TLR4 to MPLA (8, 11). This should be kept in mind when evaluating the response of human cells to AS01 containing naturally derived MPLA as opposed to formulations containing a synthetic form of MPLA such as phosphorylated hexaacylated disaccharide (PHAD) (11). Incorporation of MPLA in LPs as is the case in AS01 further reduces its reactogenicity (12, 13). QS-21 is a saponin isolated from the bark of the South American soapbark tree, *Quillaja saponaria* (14). Although QS-21 has lower toxicity than other saponin fractions, it still has hemolytic activity, limiting its direct use as a vaccine adjuvant. The hemolytic activity is caused by the high affinity of QS-21 for cholesterol leading to disruption of cell membranes. This high affinity to cholesterol, however, also allows its incorporation in cholesterol-containing LPs which eliminates the toxicity and stabilizes QS-21 (15). QS-21, which is taken up by cells via cholesterol-dependent endocytosis, stimulates both antibody and cell-mediated immune responses when administered in vaccine formulations. QS-21 alone or incorporated in LPs stimulates the release of IL-1β from human peripheral blood mononuclear cells (PBMCs), mouse macrophages, and dendritic cells primed with TLR4 agonists *in vitro* (16–18). Destabilization of lysosomes results in the release of cathepsin B, phosphorylation of Syk, and activation of the NLRP3 inflammasome (19). Although QS-21 indeed fails to release IL-1β in cells from NLRP3-deficient mice, neither CD8 T cell nor antibody responses to immunization with vaccines containing QS-21 were inhibited in the absence of NLRP3 suggesting that NLRP3 is involved in, but not essential for, the stimulatory effect of QS-21 (17, 18).

The combination of MPLA and QS-21 in liposomes to generate AS01 is thought to have synergistic effects by activating multiple signaling pathways in cells and by engaging different cell types (6). Extensive preclinical studies in mice and sheep have emphasized the effect of AS01 on innate immune cells resulting in signals that generate a robust adaptive immune response (20–24). To induce an effective immune response, AS01 and the vaccine antigen need to be injected at the same site, but do not require a physical association, at least for the gE antigen in the shingles vaccine (21). AS01 also does not enhance antigen uptake by antigen-presenting cells. Injection of AS01 in the muscle induced a rapid cytokine response at the injection site and in the draining lymph node (20, 21).

Given the differences between the human and mouse immune system (25, 26), including the previously mentioned difference in TLR4 engagement by MPLA, it is important to investigate the MoA of AS01 in humans. Preclinical studies and human clinical data support the concept that AS01 drives a robust IFN-γ response with induction of IFN-γ-related chemokines such as CXCL10 (27), but further insights may be derived from examining the effect of AS01 on human immune cells. Previous studies showed that incubation of human PBMCs with AS01 induced strong expression of TNF and IL-1β in monocytes and, to a lesser extent, dendritic cells (28). However, there was no production of IFN-γ in either T cells or NK cells indicating that further studies are needed to better understand the effect of AS01 in humans.

In this study, we investigated the effect of AS01 on the secretion of chemokines, cytokines and growth factors by human immune cells using a large multiplex panel. AS01 induced a robust chemokine response in human monocytic THP-1 cells and PBMCs in addition to the secretion of fewer cytokines and growth factors. Experiments with TLR4-inhibitors and LPs with individual components identified varying contributions of QS-21 and TLR4 agonists to the secretion of chemokines with a remarkable degree of synergy between the two components for the secretion of many chemokines. Our work further revealed a significant induction of IFN-β and IFN-stimulated genes by AS01 which has not been previously reported in preclinical studies.

## Materials and methods

### Cells

Human THP-1 cells (TIB-202; American Type Culture Collection, Manassas, VA, USA) were cultured in complete RPMI (RPMI 1640 supplemented with 2 mM L-glutamine, 55 μM 2-mercaptoethanol, 1× non-essential amino acids, 10 mM HEPES, 100 U/ml penicillin, 100 μg/ml streptomycin, and 0.25 μg/ml amphotericin) with 10% FBS (all from ThermoFisher Scientific, Waltham, MA, USA). HEK-Blue™ hTLR4 cells (Invivogen, San Diego, CA, USA) were maintained in DMEM supplemented with 2 mM L-glutamine, 100 U/ml penicillin, 100 μg/ml streptomycin, and 100 μg/ml normocin (Invivogen). THP1-Dual cells (InvivoGen) were cultured in complete RPMI supplemented with 10% FBS, 10 mg/ml blasticidin, 100 µg/ml zeocin (Invivogen), and 100 µg/ml normocin. Human peripheral blood mononuclear cells (PBMCs) pooled from five donors were purchased from HumanCells Biosciences (Milpitas, CA, USA).

### Adjuvants

AS01_B_ from Zoster Vaccine Recombinant, Adjuvanted SHINGRIX™ purchased from Myonex (Horsham, PA, USA) was used in all experiments except for the liposome variants study. Liposome variants with component ratios similar to AS01 were synthesized to assess the potential synergy between the TLR4 agonist and QS-21. These LPs were formulated through a classic thin-film hydration extrusion process as described elsewhere (29) using cholesterol (Avanti Research, Alabaster, AL, USA), 1, 2-dioleoyl-sn-glycero-3-phosphocholine (DOPC; Avanti Research), phosphorylated hexaacyl disaccharide (PHAD; Avanti Research), and QS-21 (Desert King, Chula Vista, CA, USA).

### Cell stimulation

THP-1 cells (5 x 10^5^ cells/mL) and PBMCs (2 x 10^6^ cells/mL) were seeded in 96 or 24 well plates in cell growth media and incubated with the indicated dilutions of AS01, liposome variants, or LPS (from *E. Coli* 0111:B4; Invivogen) at 100 ng/mL. In some experiments, cells were pre-incubated with 10 µM TAK-242 (SelleckChem, Houston, TX, USA), 10 µg/mL neutralizing anti-human TLR4 antibody (clone W7C11, Invivogen) or 20 µg/mL neutralizing anti-human IFNAR1 (Invivogen). Supernatants were harvested after 24 h unless otherwise specified. Supernatants were immediately analyzed for TNF and IL-1β or frozen at -80 °C for later analysis with the 96-plex chemokine/cytokine assay. In other experiments cells were collected for flow cytometry analysis or RNA isolation. The HEK-Blue™ hTLR4 and THP-1 Dual reporter cells were cultured in 96 well plates and stimulated with 12.5% AS01. Supernatants were aspirated after 24 h and analyzed for alkaline phosphatase and luciferase activity.

### Cytokine concentration measurements

Supernatants were analyzed for the concentration of TNF and IL-1β by ELISA (human TNF-α DuoSet and human IL-1β/IL-1F2 DuoSet, R&D Systems, Minneapolis, MN, USA). Supernatants were analyzed for a panel of 96 chemokines, cytokines and growth factors (Human Cytokine/Chemokine 96-Plex Discovery Assay^®^ Array (HD96), Eve Technologies, Calgary, AB, Canada). Cytokine concentrations below the minimal detection limit were given a value equal to the lowest concentration of the standards and concentrations above the maximal detection limit were given a value equal to the highest concentration of the standards.

### Quantitative reverse transcription-polymerase chain reaction

THP-1 cells were collected following incubation with AS01 or medium control for RNA extraction. Total RNA was extracted using the Quick-RNA Miniprep Kit (Zymo Research, Irvine, CA, USA) according to the manufacturer’s protocol. RNA concentration and purity were assessed by spectrophotometry (NanoDrop One, Thermo Scientific). Complementary DNA (cDNA) was synthesized from 0.5 µg of total RNA using the iScript Reverse Transcription Supermix kit (BioRad, Hercules, CA, USA) following the manufacturer’s instructions. Gene expression was quantified by RT-qPCR using PowerUp SYBR Green Master Mix (Thermo Fisher Scientific) on a QuantStudio 3 Real-Time PCR System (Thermo Fisher Scientific). qPCR data were analyzed using the comparative Cq (ΔΔCq) method as described by Livak and Schmittgen (30). Cq values of target genes were normalized to the geometric mean of the reference housekeeping genes *ACTB* (β-actin) and *GAPDH* (glyceraldehyde 3-phosphate dehydrogenase) to obtain ΔCq values. Relative expression levels were calculated as 2^-ΔΔCq^ with expression in the indicated control condition set to 1.0. Primer sets used for qRT-PCR are listed in **Supplementary Table 5**.

### Western blotting

Following stimulation with AS01 or medium only, THP-1 cells were collected and washed once with ice-cold phosphate-buffered saline (PBS). Cell pellets were lysed on ice in RIPA Lysis and Extraction Buffer (Thermo Fisher Scientific) with Halt™ Protease and Phosphatase Inhibitor Cocktail (Thermo Fisher Scientific). Lysates were incubated on ice for 10 min with intermittent mixing and purified by centrifugation at 15 000 × g for 15 min at 4 °C. The supernatants were collected and resuspended with 4× Laemmli sample buffer containing dithiothreitol (Bio-Rad) and heated at 95 °C for 5 min. Samples were separated by SDS-PAGE using 4–20% Mini-PROTEAN TGX precast gels (Bio-Rad). Proteins were then transferred onto nitrocellulose membranes using a Trans-Blot Turbo transfer system with Trans-Blot Turbo transfer packs (Bio-Rad) according to the manufacturer’s protocol. Membranes were blocked for 1 h at room temperature in SuperBlock™ Blocking Buffer (Thermo Fisher Scientific) and incubated overnight at 4 °C with primary antibodies diluted in blocking buffer. The following primary antibodies were used: phospho-STAT1 (Tyr701) (Cell Signaling Technology, Danvers, MA, USA, Cat. No. 7649), STAT1 (Cell Signaling Technology, Cat. No. 9172), phospho-STAT6 (Cell Signaling Technology, Cat. No. 9364), STAT6 (Cell Signaling Technology, Cat. No. 5397) at 1:1000 dilution and GAPDH (Cell Signaling Technology, Cat. No. 2118) at 1:2000 dilution. After primary incubation, membranes were washed three times with Tris Buffered Saline (TBS)-Tween 20 and incubated for 1 h at room temperature with HRP-conjugated anti-rabbit IgG secondary antibody (Cell Signaling Technology, Cat. No. 7074). The membranes were washed again for 3 times with TBS-Tween 20 for 10 min and subsequently developed with Clarity Max Western ECL substrate (Bio-Rad) and captured using an Syngene G:Box chemi imager (Fisher Scientific). Quantification of the western blots was performed with Image Lab 6.1 software (Bio-Rad).

### Flow cytometry

THP-1 cells were transferred to a 96-well round bottom tissue culture treated plate (CELLTREAT Scientific Products, Ayer, MA, USA) at 0.5 x 10^6^ cells/mL in 200 μL cell staining buffer (BioLegend, San Diego, CA, USA). The cells were first stained with Zombie Red Fixable Viability Dye (BioLegend). To prevent nonspecific antibody binding, cells were then incubated with Human Fc Receptor Blocking Solution (BioLegend) for 10 min at room temperature prior to antibody labeling. Cells were incubated with fluorochrome-conjugated antibodies specific to various surface markers for 45 min at 4 °C in the dark. Antibodies used are listed in **Supplementary Table 6**. After labeling, cells were washed and fixed in 4% paraformaldehyde prior to acquisition. Data were acquired on an Attune NxT Flow Cytometer (Thermo Fisher Scientific). Compensation was performed using single-stained UltraComp eBeads (Thermo Fisher Scientific) for each fluorochrome according to the manufacturer’s instructions. Gates were defined using fluorescence minus one (FMO) controls where appropriate. The data was analyzed using FlowJo software (FlowJo, Ashland, OR, USA). Median fluorescence intensity (MFI) and percentage of positive cells were quantified.

### Statistical analysis

The statistical significance of differences between experimental groups was determined by one-way analysis of variance (ANOVA) test followed by a Tukey’s multiple comparisons test. The differences in mRNA expression were analyzed by converting the fold change into log10 values, followed by two-way ANOVA and Sidak’s multiple comparison test. Statistical analyses were performed with GraphPad Prism version 10.4.1 (GraphPad Software, San Diego, CA, USA) for Windows.

## Results

### Determination of optimal AS01 stimulation conditions

The adjuvant AS01 comes in two forms, AS01_B_ containing 100 µg/mL of MPLA and 100 µg/mL QS-21, and AS01_E_ with half those concentrations (6). We used AS01_B_ in our studies and refer to it throughout this manuscript as AS01. Previous publications have reported a wide range of concentrations to evaluate the effect of AS01 on human immune cells ranging from a 1:100 dilution to a 1:4 dilution (i.e., 1.0 µg/mL to 25 µg/mL MPLA and QS-21) (28, 31, 32). We therefore first determined the effect of serial dilutions of AS01 on THP-1 cells using the concentration of TNF and IL-1β in 24 h supernatants as markers of cell stimulation. AS01 induced secretion of TNF in a dose-dependent manner with the highest concentration observed at 50% AS01 (**Figure 1a**). Incubation with AS01 also caused a dose-dependent, but very limited release of IL-1β (**Figure 1b**). Importantly, exposure of THP-1 cells to AS01 did not induce significant cell membrane damage as indicated by the absence of LDH release even at the highest concentration of 50% (**Figure 1c**). In addition to the AS01 concentration, we also evaluated the effect of the incubation time on TNF secretion induced by AS01 and 24 h yielded the maximum production (**Figure 1d**). Based on these data and practical considerations, we chose 24 h stimulation with 12.5% AS01 as the assay conditions.

**Figure 1:**
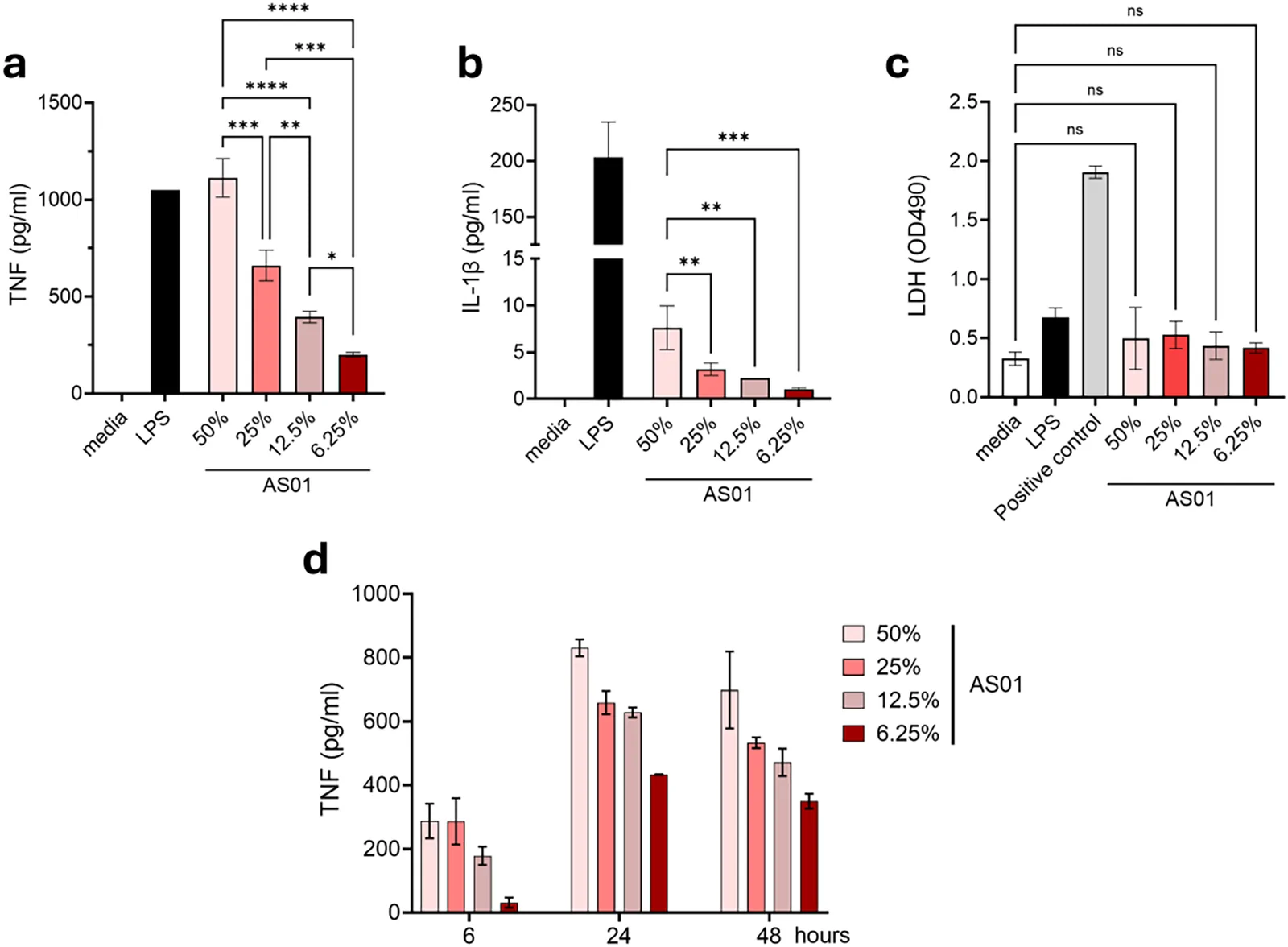
Effect of concentration of AS01 and incubation time on THP-1 cells. **(a–c)** THP-1 cells were incubated for 24 h with 100 ng/mL LPS or serial dilutions of AS01 starting from 50% (contains 50 µg/mL MPLA and 50 µg/mL QS-21) and the supernatants were analyzed for TNF **(a)**, IL-1β **(b)** and LDH **(c)**. **(d)** Concentration of TNF in the supernatants of THP-1 cells stimulated with serial dilutions of AS01 for 6, 24 or 48 hours. Bars indicate the mean ± SD of triplicate wells. *p < 0.05; **p < 0.01; ***p < 0.001; ****p < 0.0001.

### AS01 induced a broad chemokine response and limited cytokine response from THP-1 cells and primary blood leukocytes

Using the optimized culture conditions, THP-1 cells were incubated with 12.5% AS01 and supernatants were evaluated by multiplex assay to measure the concentration of 30 chemokines and 66 cytokines and growth factors (full list of chemokines, cytokines and growth factors see **Supplementary Table 1**). AS01 significantly increased the secretion of 10 chemokines from about two-fold for CXCL12 to nearly 1, 000-fold for CCL4 compared to medium controls (**Figure 2a**; **Supplementary Table 2**). AS01 increased the secretion of six out of 66 cytokines and growth factors (**Figure 2b**; **Supplementary Table 2**) and the concentrations were relatively low with only the concentrations of IL-1RA and TNF exceeding 100 pg/ml.

**Figure 2:**
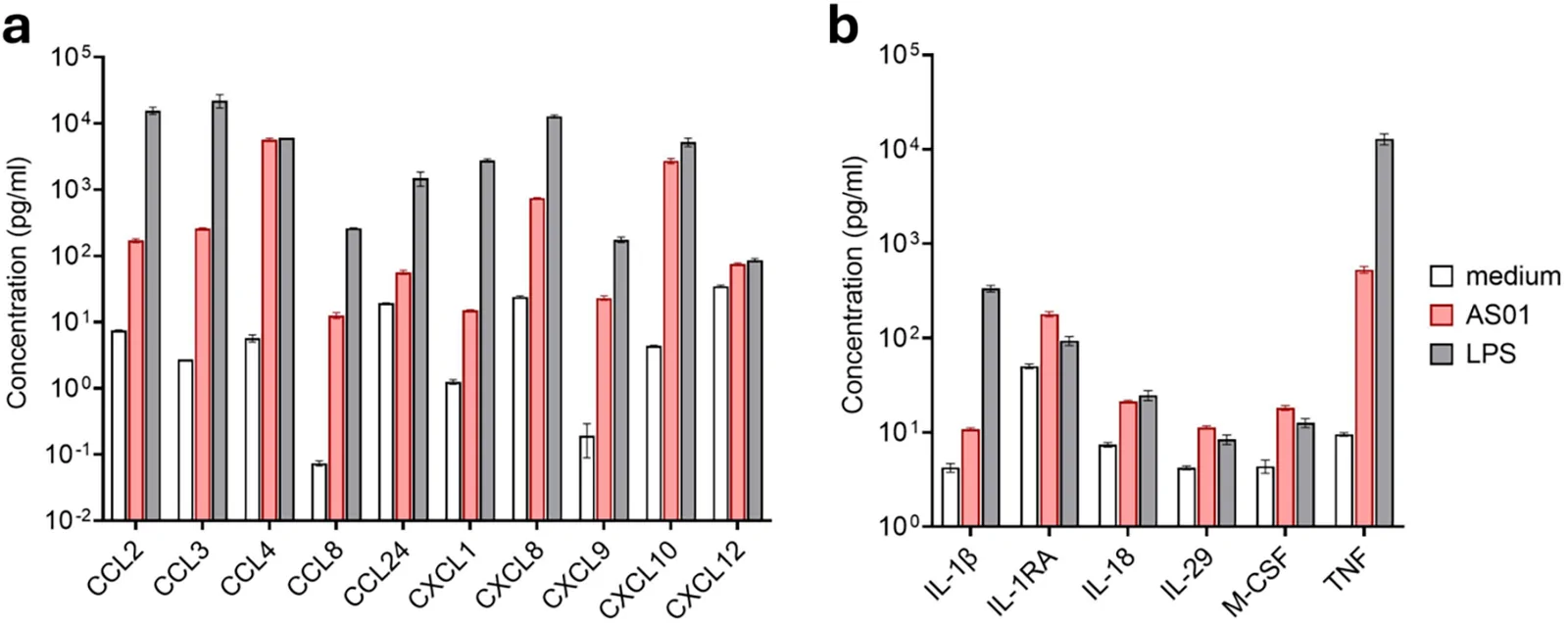
Chemokine and cytokine secretion from THP-1 cells stimulated with AS01. THP-1 cells were incubated for 24 h with medium, 12.5% AS01 (12.5 µg/mL MPLA and QS-21), or LPS (100 ng/mL) and the concentrations of chemokines and cytokines were determined. The graphs show the chemokines **(a)** and cytokines or growth factors **(b)** that were significantly increased by AS01 compared with the medium control at p < 0.01 and had a concentration > 10 pg/mL in AS01 supernatants. The bars indicate the mean ± SD of triplicate wells.

To confirm the ability of AS01 to induce chemokine and cytokine secretion in primary human immune cells, PBMCs pooled from five healthy donors (to avoid donor-to-donor variability) were incubated with medium, AS01, or LPS and 24 h supernatants were collected for 96-plex chemokine/cytokine analysis. While there was no measurable chemokine or cytokine production from cells incubated with media as control, there was again a robust induction of chemokines by AS01 with a significant increase (at p < 0.01) in 15 of the 30 chemokines tested (**Figure 3a**; **Supplementary Table 3**). Similar to THP-1 cells, AS01 induced increased secretion of CCL3, CCL4, CCL8, CXCL1, CXCL8 and CXCL11 by PBMCs. In addition, AS01-stimulated PBMCs secreted CCL1, CCL7, CCL13, CCL20, CCL22, CCL23, CCL28, CXCL5 and CXCL6, although the increases were modest compared with the medium control. AS01 increased secretion of 12 cytokines and growth factors (**Figure 3b**; **Supplementary Table 3**), three of which (IL-1β, IL-1RA, TNF) were also induced in THP-1 cells. There was a marked induction of the pro-inflammatory cytokines IL-1α, IL-1β, IL-6 and TNF as well as anti-inflammatory cytokines, in particular IL-1RA and IL-10.

**Figure 3:**
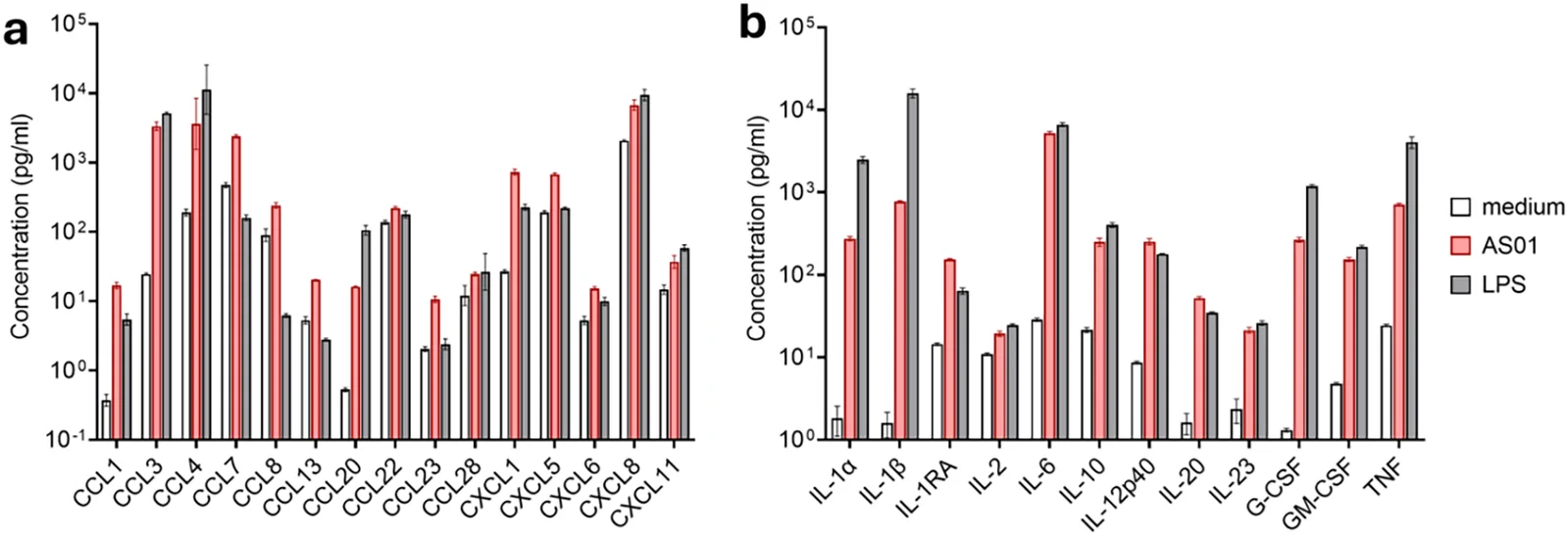
Chemokine and cytokine secretion by human PBMCs stimulated with AS01. Pooled PBMCs were incubated for 24 h with medium, 12.5% AS01 (12.5 µg/mL MPLA and QS-21), or LPS (100 ng/mL) and the concentrations of chemokines and cytokines were determined. The graphs show the chemokines **(a)** and cytokines or growth factors **(b)** that were significantly increased by AS01 compared with the medium control at p < 0.01 and had a concentration > 10 pg/mL in AS01 supernatants. The bars indicate the mean ± SD of triplicate wells.

### AS01 engages the TLR4 receptor in THP-1 cells

To determine the role of TLR4 signaling in the response to AS01, THP-1 cells were incubated with the TLR4-antagonist TAK-242 prior to the addition of AS01. Supernatants were then analyzed by multiplex assay for the same panel of 96 chemokines, cytokines, and growth factors. The degree of inhibition of chemokine and cytokine secretion by TAK-242 varied from 0 to 88.3% (**Figure 4a**). The partial inhibition of TNF secretion by interference of TLR4 signaling from TAK-242 was confirmed with an anti-TLR4 antibody (**Figure 4b**). These experiments indicate a role for TLR4 signaling in the immunostimulatory activity of AS01. To further validate these findings, the effect of AS01on reporter cell lines was determined. AS01 activated a human TLR4 reporter cell line (HEK-Blue™ hTLR4) and activated both NF-κB and Interferon Regulatory Factor (IRF) signaling pathways in THP-1 dual reporter cells, although at a much lower level than LPS (**Figures 4d–f**).

**Figure 4:**
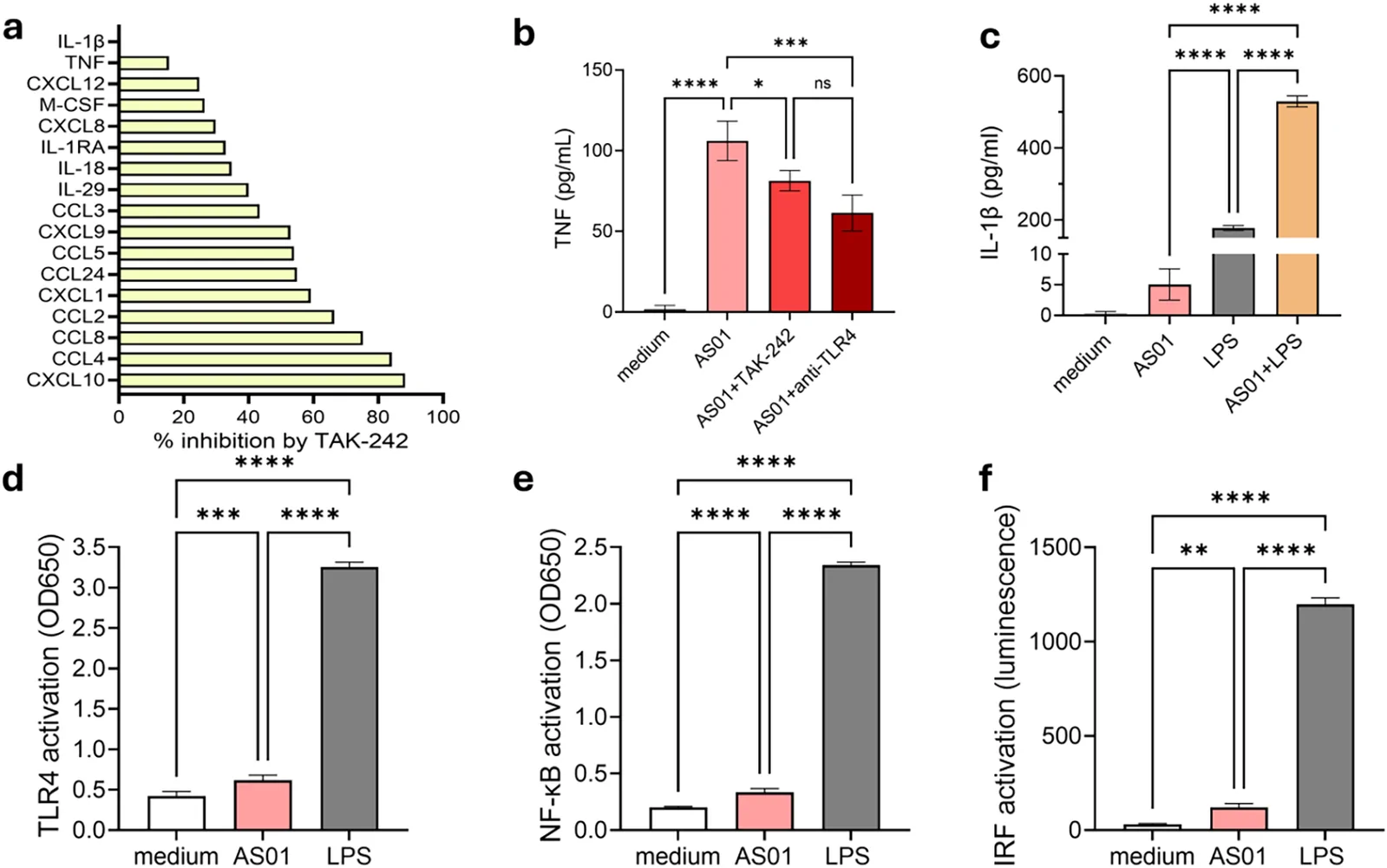
Role of TLR4 signaling in AS01-induced chemokine and cytokine secretion by THP-1 cells. **(a)** Effect of the TLR4-inhibitor TAK-242 on the secretion of chemokines and cytokines. **(b)** Effect of the TLR4 inhibitor TAK-242 and an anti-TLR4 antibody on TNF secretion by THP-1 cells. **(c)** Effect of priming of THP-1 cells with LPS on AS01-induced IL-1β release into the supernatant. **(d)** TLR4 signaling induced by AS01 and LPS in a human TLR4 HEK-Blue™ reporter cell line. **(e, f)** Activation of NF-κB **(e)** and IRF **(f)** signaling pathways induced by AS01 and LPS in THP-1 Dual™ cells. Bars indicate the mean ± SD of three to six replicate wells. *p < 0.05; **p < 0.01; ***p < 0.001; ****p < 0.0001; ns, not significant.

The induction of IL-1β secretion is a two-step process (33, 34). Cells typically need to be primed to increase the expression of NLRP3, pro-caspase-1 and pro-IL-1β, which can be achieved by activation of TLRs or binding of TNF. The second step induces the assembly of the NLRP3 inflammasome with active caspase-1 which cleaves pro-IL-1β into the biologically active form of IL-1β that is then released from the cell. Previous work has shown that QS-21 in LPs can induce NLRP3 inflammasome activation (16–18), but the small amount of IL-1β in supernatants of THP-1 cells exposed to AS01 suggests that the TLR4 activation by MPLA was not sufficient to prime the cells. Indeed, priming the cells with LPS followed by the addition of AS01 significantly increased the IL-1β release from THP-1 cells (**Figure 4c**). Taken together, these data indicate that TLR4 signaling contributes to the response of THP-1 cells to AS01, but that AS01 is a weak stimulator of TLR4.

### Synergy of QS-21 and TLR4 agonists in LPs

The previous experiments support a role for both QS-21 and the TLR4 agonist MPLA in the activation of THP-1 cells. To further delineate how QS-21 and engagement of the TLR4 signaling pathway contribute to the production of chemokines and cytokines, LPs were synthesized as empty particles, with QS-21, with phosphorylated hexa-acyl disaccharide (PHAD, a synthetic version of MPLA), or containing both immunomodulators. Incubation of THP-1 cells with empty LPs did not induce a significant secretion of chemokines and cytokines compared with medium only (not shown). The combination of QS-21 and PHAD in LPs induced a broad chemokine response and a limited cytokine response similar to the results with AS01. Incubation of THP-1 cells with LP-QS-21 or LP-PHAD induced secretion of chemokines and cytokines, but at a lower concentration than the combination. Compared with LP-PHAD, LP-QS-21 induced more chemokines CCL2 (p < 0.001), CCL3 (p < 0.01), CCL8 (p < 0.001), CCL24 (p < 0.01), CXCL8 (p < 0.001), and CXCL12 (p < 0.001), as well as IL-1RA (p < 0.001), IL-29 (p < 0.001), PDGF-AA (p < 0.001) and TNF (p < 0.001), whereas LP-PHAD induced more IL-12p40 than LP-QS-21 (p < 0.001). Synergistic effects, defined here as a significantly increased concentration of the chemokine or cytokine in supernatants of LP-QS-21/PHAD versus the sum of the concentrations of LP-QS-21 and LP-PHAD, were observed for CCL2, 3, 4, 8, CXCL1, 8, 9, 10, 11, and TNF (p < 0.05). A notable synergistic effect was seen for CCL4 in which the combination of both immunostimulators induced a ~40-fold greater secretion than the individual components in LP (**Figure 5**; **Supplementary Table 4**).

**Figure 5:**
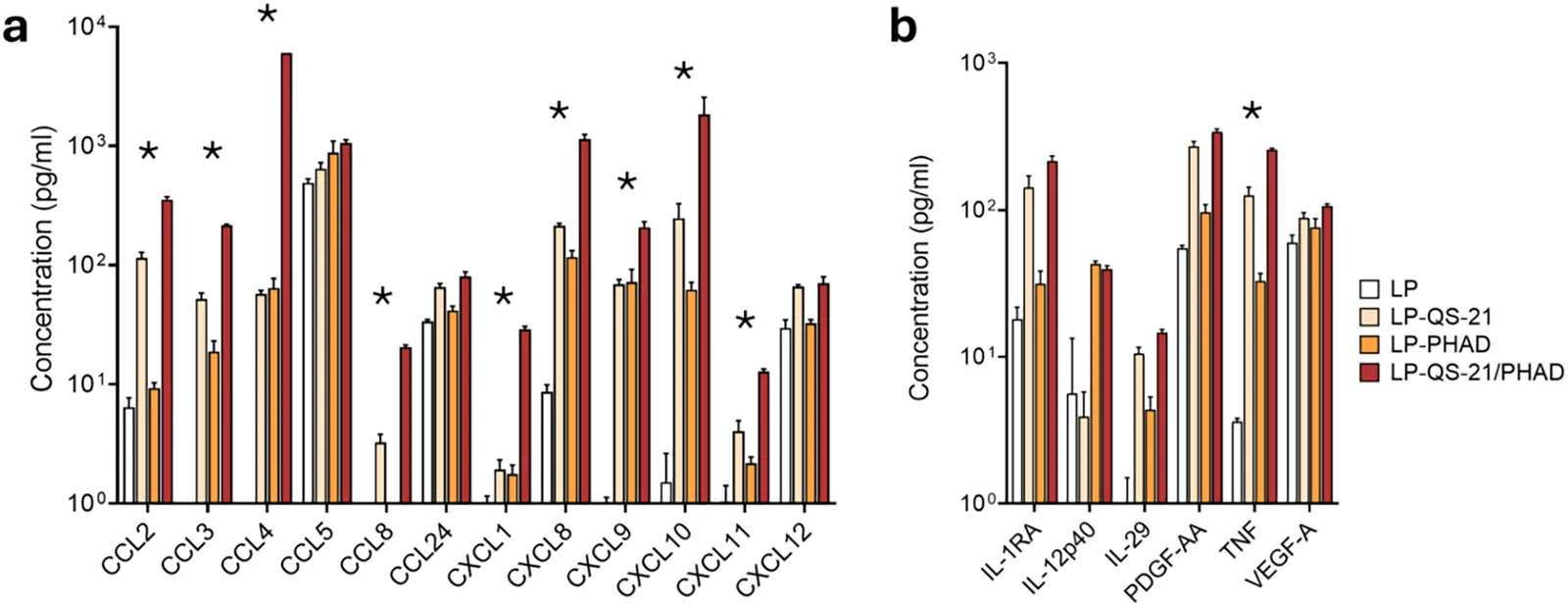
Synergistic effect of QS-21 and PHAD on chemokine and cytokine secretion. THP-1 cells were incubated for 24 h with empty LPs, LP-QS-21 (18.75 µg/mL QS-21), LP-PHAD (18.75 µg/mL PHAD), or LP-QS-21/PHAD (18.75 µg/mL each). The graphs show the concentrations of chemokines **(a)** and cytokines and growth factors **(b)** in the supernatants that were significantly increased by incubation with LP-QS21/PHAD compared with LP only at p < 0.01 and had a concentration > 10 pg/mL. The bars indicate the mean ± SD of triplicate wells. *indicates synergistic effects of QS-21 and PHAD in inducing chemokine or cytokine secretion.

### AS01 induces type I interferon and interferon-stimulated genes in THP-1 cells

The previous results (**Figure 4f**) showed that AS01 activates the Interferon Regulatory Factor (IRF) signaling pathway in THP-1 cells and induced the secretion of CXCL9, CXCL10 and CXCL11 which are known as interferon (IFN)-inducible chemokines in THP-1 cells and PBMCs. However, we did not observe an increased concentration of either type I IFNs (IFN-α2, IFN-β and IFN-ω) or IFN-γ in the supernatants of THP-1 cells and PBMCs stimulated with AS01. As type I IFNs act in an autocrine and paracrine manner, binding to its receptor may preclude an increase of unbound IFN in the supernatant. We therefore investigated the transcription of IFN-stimulated genes (ISGs) by quantitative RT-PCR (primers used for qRT-PCR see **Supplementary Table 5**). There was increased mRNA expression of the antiviral ISGs *IFIH1, ISG15*, *MX1*, and *OAS1* at 8 h and more so at 24 h after stimulation with AS01 (**Figure 6a**). In addition, the expression of genes encoding for STAT1, IFN-β, IFN-γ, and the transcription factor IRF7 was significantly increased (**Figure 6a**). AS01 not only increased expression of STAT1 but also induced phosphorylation of STAT1 (**Figure 6b**). This was specific for STAT1 as no phosphorylation of STAT6 was observed. These data suggest a role for not only IFN-γ, which was previously identified in mouse studies (20), but also type I IFNs in the immunostimulatory effect of AS01. To further investigate the role of type I IFN, we incubated THP-1 cells with an anti-human type I interferon receptor subunit 1 (IFNAR1) antibody, anifrolumab, that inhibits type I IFN signaling, before the addition of AS01. Similar to the data shown in **Figure 6**, AS01 induced marked upregulation of antiviral ISGs as well as *STAT1*, *CXCL10*, *IFNB*, *IFNG* and *IRF7*. Blocking of the IFN receptor, greatly reduced the expression of most antiviral ISGs with exception of *IFIH1*, and also reduced *IFNG*, *CXCL10* and *IRF7* mRNA (**Figure 7**). Inhibition of signaling through the type I IFN receptor did not reduce the expression of *IFNB* mRNA, suggesting that its expression is a direct effect of AS01. Together, these data reveal that AS01 induces the expression of type I IFN and this may contribute to the immunostimulatory effect of AS01.

**Figure 6:**
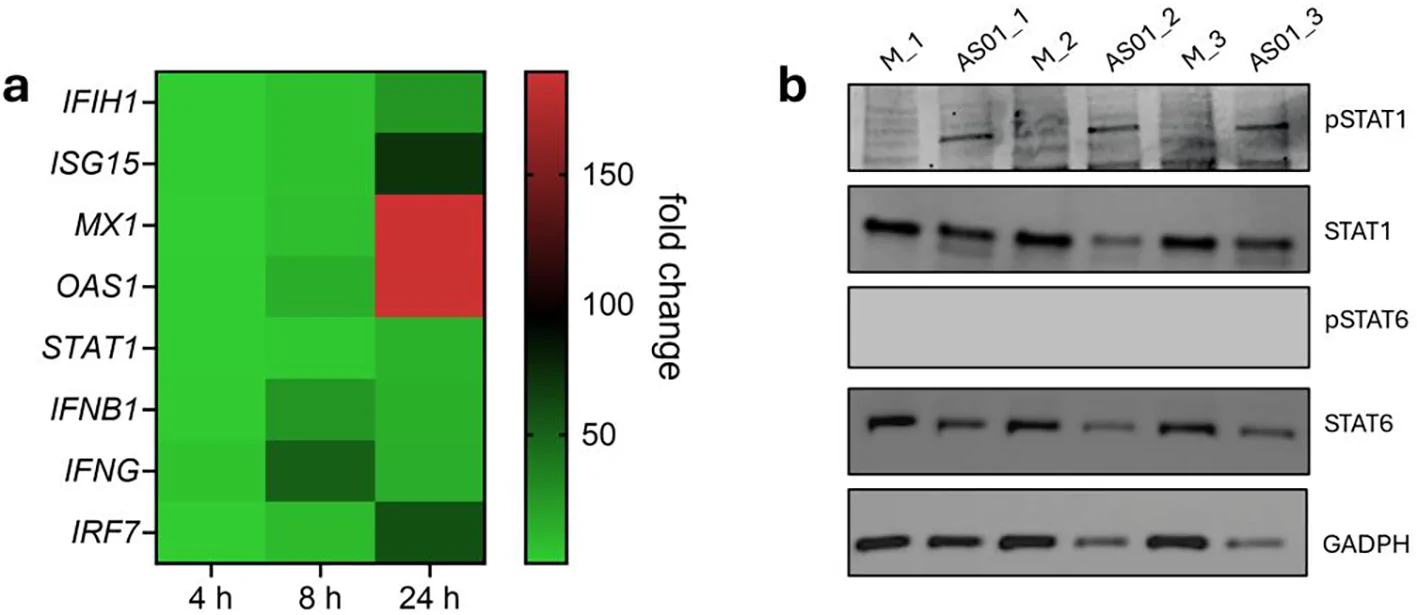
AS01 induces increased expression of type I IFN and IFN-stimulated genes and induces IFN-signaling in THP-1 cells. **(a)** THP-1 cells were incubated for 4,8 or 24 h with AS01 and RNA was isolated from the cells and processed for quantitative RT-PCR. Relative gene expression was calculated compared to unstimulated THP-1 cells using the 2^-ΔΔCq^ method. The heatmap colors represent the mean fold change of two independent experiments with three replicates each. **(b)** Western blot analysis for STAT 1 and 6 and phosphorylated STAT1 and STAT6. THP-1 cells were incubated for 8 h with medium (M) or AS01 followed by protein extraction. Data are shown from three independent cell culture experiments.

**Figure 7:**
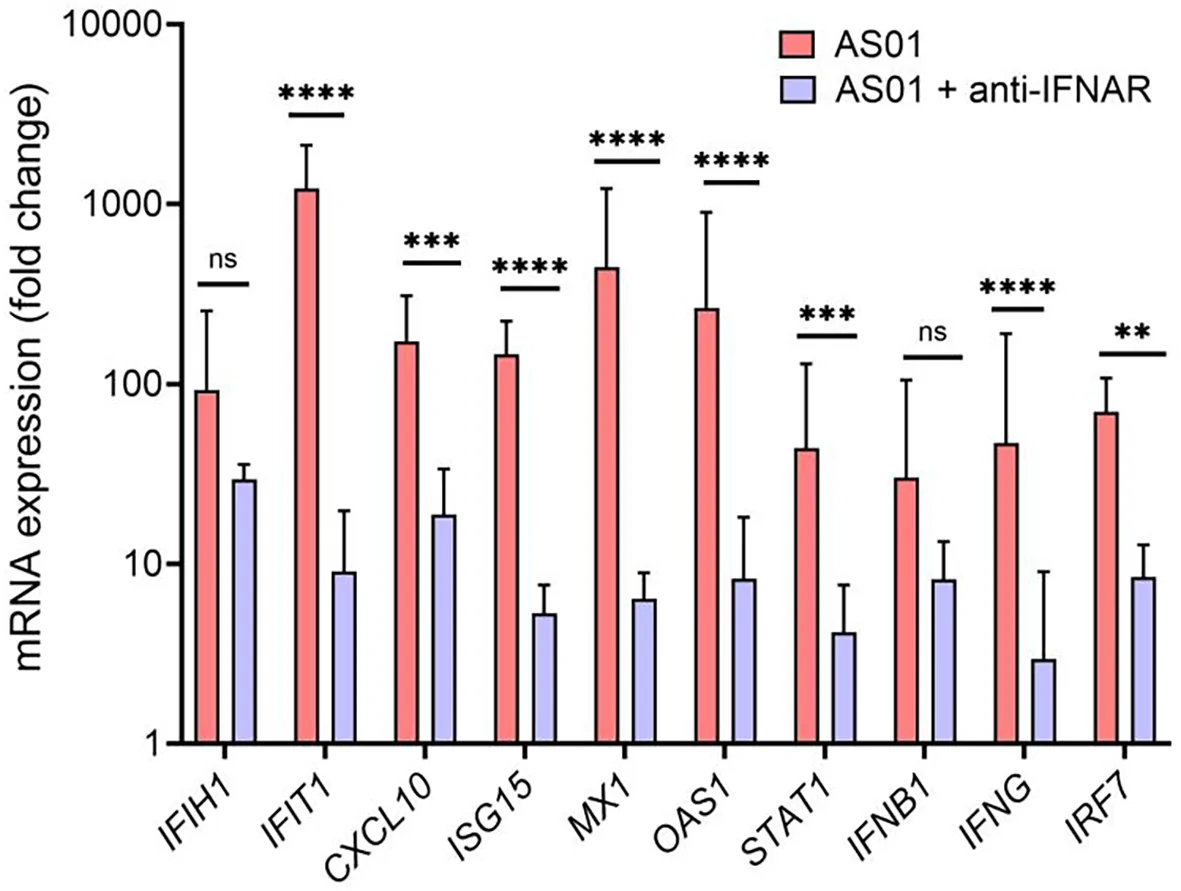
The increased expression of IFN-stimulated genes induced by AS01 in THP-1 cells is reduced by blocking of the type I IFN receptor. THP-1 cells were incubated with an anti-IFNAR1 antibody prior to stimulation with AS01. After 24 hours, RNA was isolated from the cells and processed for quantitative RT-PCR. Relative gene expression was calculated compared to unstimulated THP-1 cells using the 2^-ΔΔCq^ method. **p < 0.01; ***p < 0.001; ****p < 0.0001; ns, not significant.

### AS01 induces the expression of the DC maturation marker CD83 on THP-1 cells

Besides the effect of AS01 on the secretion of chemokines, cytokines and growth factors, we investigated its effect on the phenotype of THP-1 cells, specifically the expression of costimulatory molecules. Incubation of THP-1 cells with LPS for 24 h increased the expression of CD40, CD54, and CD83, with no significant effect on the expression of CD80 and CD86 (**Figure 8**). AS01 and LPs formulated with QS-21 and PHAD did not alter the expression of CD40, CD54, CD80 and CD86, but increased the expression of CD83 to a higher level compared with LPS (**Figure 8**). The expression of CD83 induced by LP-QS-21 was similar to that induced by AS01 and LP-QS-21/PHAD, and greater than that induced by LP-PHAD or LPS. This suggests that, while TLR4-signaling enhances CD83 expression, QS-21 is the major driver in the AS01-induced CD83 expression in THP-1 cells.

**Figure 8:**
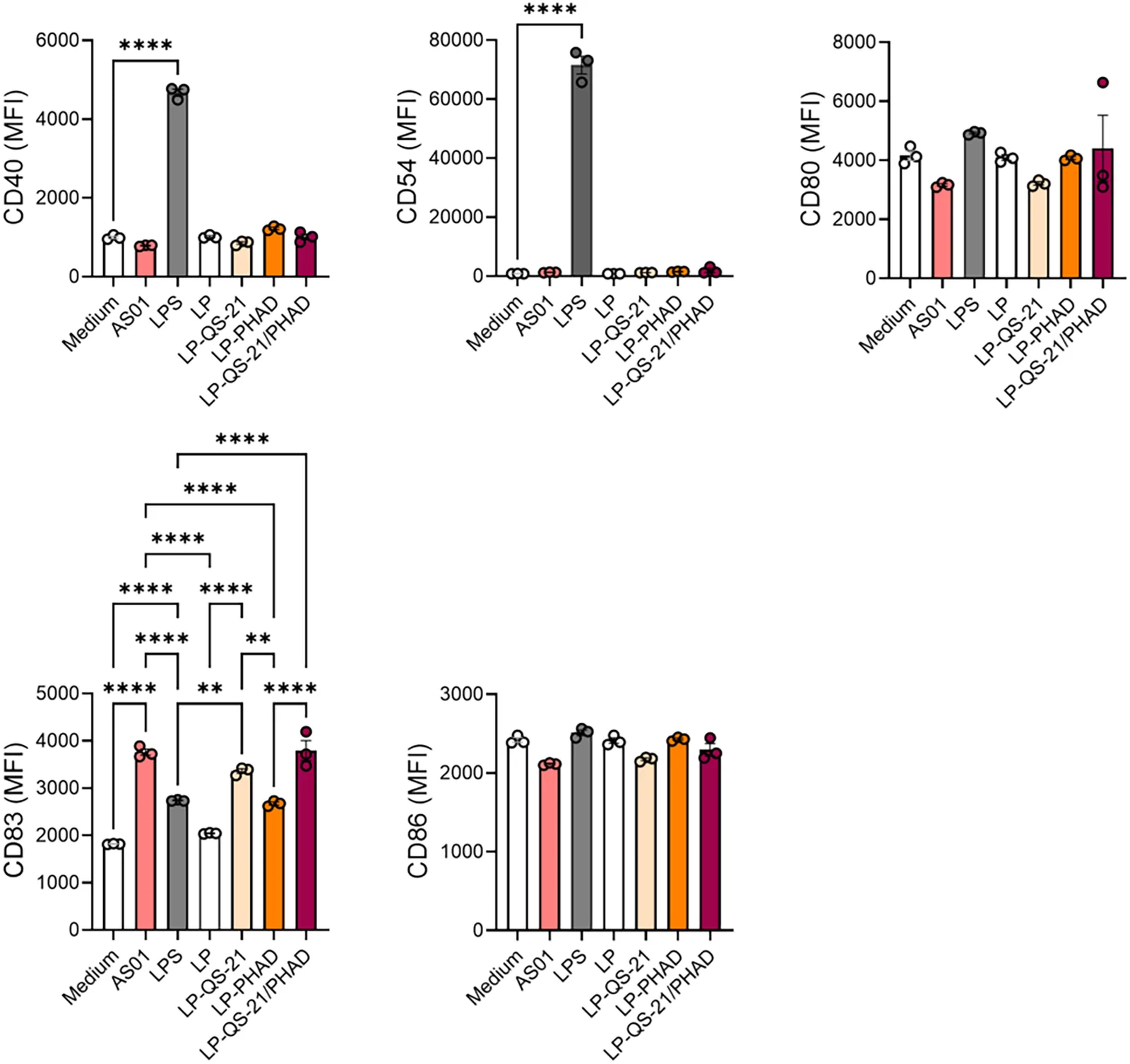
Effect of AS01 on the phenotype of THP-1 cells. Cells were unstimulated (Medium) or incubated with AS01 or LPs for 24 h. The expression levels of costimulatory molecules including CD40, CD54, CD80, CD83, and CD86 were assessed by flow cytometry. The bars indicate the mean ± SD of three replicates. *p < 0.05; **p < 0.01; ***p < 0.001; ****p < 0.0001.

## Discussion

The AS01 adjuvant was developed to utilize the potential synergy between two known immunostimulators, MPLA and QS-21, delivered together to antigen-presenting cells in the form of LPs (6, 35). Preclinical studies have demonstrated synergistic effects of QS-21 and MPLA in enhancing CD4^+^ and CD8^+^ T cell responses, albeit with a stronger role for QS-21 than MPLA (20, 36). There was no difference in serum antibody titers between mice immunized with LP-QS-21 versus AS01 or AS01-like formulations (20, 36, 37). Cell culture experiments with human cells revealed synergistic effects of QS-21 and PHAD on the secretion of certain cytokines and the activation of CD8^+^ T cells (37). Our results support synergistic roles of QS-21 and MPLA in the induction of cytokine secretion by THP-1 cells and further demonstrate that the contribution of each component varied and was cytokine-dependent.

Monocytes respond to immunopotentiators by the secretion of chemokines and cytokines that in turn recruit and activate other immune cells resulting in inflammation and initiation of the adaptive immune response. The NF-κB and IRF signaling pathways are two major signaling pathways involved in the transcription of chemokine and cytokine genes. AS01 induced activation of both pathways as indicated by the experiments with THP-1 Dual cells and this is also reflected in the robust secretion of a broad panel of chemokines and a more modest number of cytokines. The strong induction of chemokine secretion is consistent with the rapid accumulation of neutrophils, monocytes and dendritic cells at the injection site following intramuscular injection of AS01 in mice (21). These cells all participate in the uptake of vaccine antigen and transport to the draining lymph node as demonstrated in experiments with cannulated sheep (23). The NF-κB transcription factors control the production of cytokines like TNF and the chemokines CXCL1 and CXCL8 which attract neutrophils via chemokine receptors CXCR1 and CXCR2, and CCL2, CCL3, CCL4, CCL5, and CCL8 which attract lymphocytes, monocytes, macrophages and dendritic cells via chemokine receptors CCR1, CCR2, and CCR5 (38, 39). On the other hand, CXCL9, CXCL10, and CXCl11, which attract CD4^+^ and CD8^+^ T cells and NK cells via CXCR3 (38, 39) are associated with the IRF signaling pathway. Among the nine IRFs identified in human cells, IRF3 is constitutively expressed. Activation of IRF3 leads to the transcription and secretion of IFN-β which binds to the IFN receptor and induces Interferon Stimulated Genes (ISGs) via JAK-STAT signaling. One of the ISGs is IRF7 which contributes to positive feedback and amplification of the ISG expression (40, 41). In addition, a subset of ISGs can be induced directly by IRF3 without the involvement of the IFN-receptor (42, 43). It has recently been suggested that MPLA does not efficiently activate the IRF3 pathway and does not induce type I IFN in human cells (6). However, this is not consistent with the literature as both naturally derived MPLA and synthetic PHAD induce IFN-β and interferon-stimulated genes in human dendritic cells (11). We did not observe an increased concentration of type I IFNs in the supernatant of THP-1 cells or PBMCs following incubation with AS01. Similarly, AS01 did not induce an increase of IFN-α in cultures of human lymph node slices (31). Studies have shown that secreted IFNs are rapidly bound by their receptors as they act primarily in an autocrine and paracrine fashion to induce the expression of interferon-stimulated genes (ISGs) which play important roles in limiting viral replication (44, 45). Indeed, we observed increased expression of *IFNB mRNA* as well as the antiviral ISGs *IFIH1, IFIT1*, *ISG15*, *MX1*, and *OAS1* in THP-1 cells stimulated with AS01. The increased expression of ISGs was inhibited by blocking the type I IFN receptor, confirming that this response is driven by AS01-induced type I IFN. Type I IFNs not only activate antiviral mechanisms as part of the innate immune response to viral infections but also enhance the adaptive immune responses. They stimulate dendritic cell maturation (46, 47), the expansion and differentiation of CD4^+^ and CD8^+^ T cells (48, 49), and the antibody response via direct and indirect effects on B cells (50, 51). On the other hand, transient inhibition of the type I IFN receptor at the time of vaccination increased the immune response to live attenuated viral vaccines and mRNA vaccines and enhanced the development of CD8^+^ effector and memory T cells (52–54). Together, these data suggest that induction of type I IFNs in antigen-presenting cells contributes to the immunostimulatory effect of AS01, while inhibition of type I IFN or IFN signaling at the time of vaccination may have a beneficial effect for certain vaccine applications.

Previous studies in mice have revealed rapid expression of *Ifng* mRNA in the draining lymph node following injection of AS01 and identified NK cells and CD8^+^ T cells as the main cellular sources of the early IFN-γ response (20). The concentration of IFN-γ in serum of humans and nonhuman primates increased following injection of an AS01-adjuvanted vaccine (20), and the production of IFN-γ and IFN-γ-related chemokines such as CXCL9 and CXCL10 is considered a “hallmark of the AS01 innate signature” (6). Furthermore, incubation of human lymph node slices with AS01 or an AS01-like adjuvant induced increased secretion of IFN-γ (31, 55) with NK cells as the main source (55). We did not observe a significant increase of IFN-γ in supernatants of THP-1 cells or PBMCs incubated with AS01. However, AS01 increased the expression of *IFNG* mRNA in THP-1 cells. Although T cells and NK cells are generally considered the main sources of IFN-γ, antigen-presenting cells are another potential source of this cytokine (56). Blocking the type I IFN receptor reduced the expression of *IFNG* mRNA indicating that the response is induced by type I IFN as shown previously for human dendritic cells (57).

The greatest fold increase in chemokine secretion in THP-1 cells and PBMCs was measured for CCL3 and CCL4, chemokines that preferentially attract CD4 and CD8 T cells (58). Previous studies have shown that PBMCs and isolated peripheral blood monocytes secrete CCL4 upon stimulation with the TLR agonists poly(I:C), LPS and R848 (59) and with the squalene emulsion adjuvant MF59 (60), but not with an aluminum hydroxide adjuvant (60). Depletion of monocytes from PBMCs abolished the secretion of CCL4 following stimulation with MF59, suggesting a negligible contribution of other cell types (60). In contrast, incubation of whole blood cultures and PBMCs with the TLR7/8 agonist R848 increased the number of CCL4^+^ cells as determined by intracellular cytokine staining, but the largest proportion of CCL4^+^ cells were neutrophils in whole blood cultures and NK cells among PBMCs rather than monocytes (61). While the level of CCL4 secretion by neutrophils and NK cells relative to monocytes was not determined, this finding suggests that cells other than monocytes can contribute to the production of CCL4 at the injection site and draining lymph node following injection of AS01 adjuvant. CCL4 was identified as a potential marker of vaccine reactogenicity in a human cell culture system of PBMCs and endothelial cells (MIMIC PTE) incubated with 10 different vaccines (62). In this study, three bacterial vaccines, but not the AS01-adjuvanted Shingrix vaccine, induced CCL4 secretion. However, the vaccines were serially diluted starting at 1:100, which is a much lower concentration of AS01 than what was used in our experiments.

In our study, AS01 induced modest activation of TLR4 as indicated by the response of HEK-Blue™ hTLR4 cells and consistent with the effect on THP-1 Dual cells. As previously reported, embedding MPLA in LPs dampens its biological activity (12, 13). Similarly, the incorporation of QS-21 in LPs reduces its activity which is important to limit the reactogenicity of adjuvants formulated with QS-21 (35). The LPs are phagocytized by monocytes which is essential for the ability of QS-21 to activate the cells (19). QS-21 causes permeabilization of the lysosomal membrane which leads to activation of the NLRP3 inflammasome and the NF-κB signaling pathway (19). Thus, both MPLA and QS-21 in the AS01 formulation likely contribute to NF-κB signaling detected in HEK-Blue™ hTLR4 and THP-1 Dual™ cells. The activation of the NLRP3 inflammasome results in release of IL-1β from primed cells. However, the NF-κB signaling in THP-1 cells was insufficient to prime the cells for IL-1β secretion following treatment with AS01 and significant secretion required a priming step with LPS. In contrast, AS01 treatment alone induced marked secretion of IL-1β in PBMCs. As monocytes are the main source of IL-1β secreted by PBMCs (63), this suggests a difference in response to AS01 between THP-1 cells and human primary monocytes in the requirement of a priming step for IL-1β secretion. This is consistent with a previous report that a separate priming step is not necessary for activation of the NLRP3 inflammasome in human monocytes in contrast to THP-1 cells or macrophages (64).

Incubation of THP-1 cells with AS01 did not affect the expression of costimulatory molecules CD40, CD54, CD80 and CD86, but increased the expression of CD83. While LPS and LP-PHAD induced a modest increase of CD83 expression, LP-QS-21 increased the expression to a larger extent similar to LP-QS-21/PHAD and AS01, suggesting that the increase of CD83 is largely driven by QS-21. Increased expression of CD83 on monocytes, macrophages and dendritic cells was also reported following incubation of human lymph node slices with AS01 and AS01-like adjuvants (31, 55). CD83 is considered a marker of dendritic cells maturation (65), and its expression is necessary for proper activation of T cells by human dendritic cells (66, 67). This suggests that AS01 enhances the maturation of dendritic cells which is a critical step in the initiation of the immune response. Moreover, our data suggests that CD83 expression could be utilized as a potential biomarker for the activity of AS01 and other QS-21-containing adjuvants.

Following intramuscular injection of AS01, adjuvant rapidly reaches the draining lymph node and induces chemokine and cytokines both at the injection site and the lymph node (20, 21). We selected a concentration of 12.5% AS01 to evaluate its effect on THP-1 cells and PBMCs based on the response to different dilutions and lack of cytotoxicity. Previous studies used a wide range of concentrations, ranging from 1:100 for primary monocytes to 1:4 for human lymph node slices (28, 31). Immune cells are likely exposed to a lower concentration of AS01 in the lymph node than at the injection site and future experiments should investigate the effect of different concentrations of the adjuvant.

Our studies focused on the effect of AS01 on human THP-1 cells, a well-established model of monocytes (68). Although not identical in their biological response to certain stimuli as noted above and elsewhere (64, 69, 70), the use of THP-1 cells offers the advantage that one can obtain fairly consistent and reproducible results which makes it a useful model to investigate adjuvant MoA and a high throughput system to screen potential new adjuvant candidates. However, the complex nature of immune responses is not captured with a single cell line. We verified some of our observations in experiments with pooled human PBMCs as a source of primary immune cells. The use of pooled cells was aimed at reducing the wide interindividual variation seen in responses of PBMCs to immunopotentiators (71, 72), but this does not address the biological variability that may occur in response to AS01-adjuvanted vaccines. In addition, the response of PBMCs in cell culture does not mimic the cellular dynamics and complexity of the draining lymph node or the inflammation at the injection site. Studies with three-dimensional human cell culture systems and organoids (73) are necessary to further investigate the MoA of AS01 and related adjuvants. Recent reports on the effect of AS01 on human lymph node slices are promising but also demonstrate the challenges such as selective survival of cell populations, limited availability of material, and donor-to-donor variation (31, 55).

In summary, we demonstrated that AS01 induces a robust chemokine response in human immune cells that relies on synergistic interactions between QS-21 and MPLA (see **Figure 9**). Our studies revealed that AS01 induced a type I IFN-associated transcriptional profile that is partially dependent on signaling via the type I IFN receptor.

**Figure 9:**
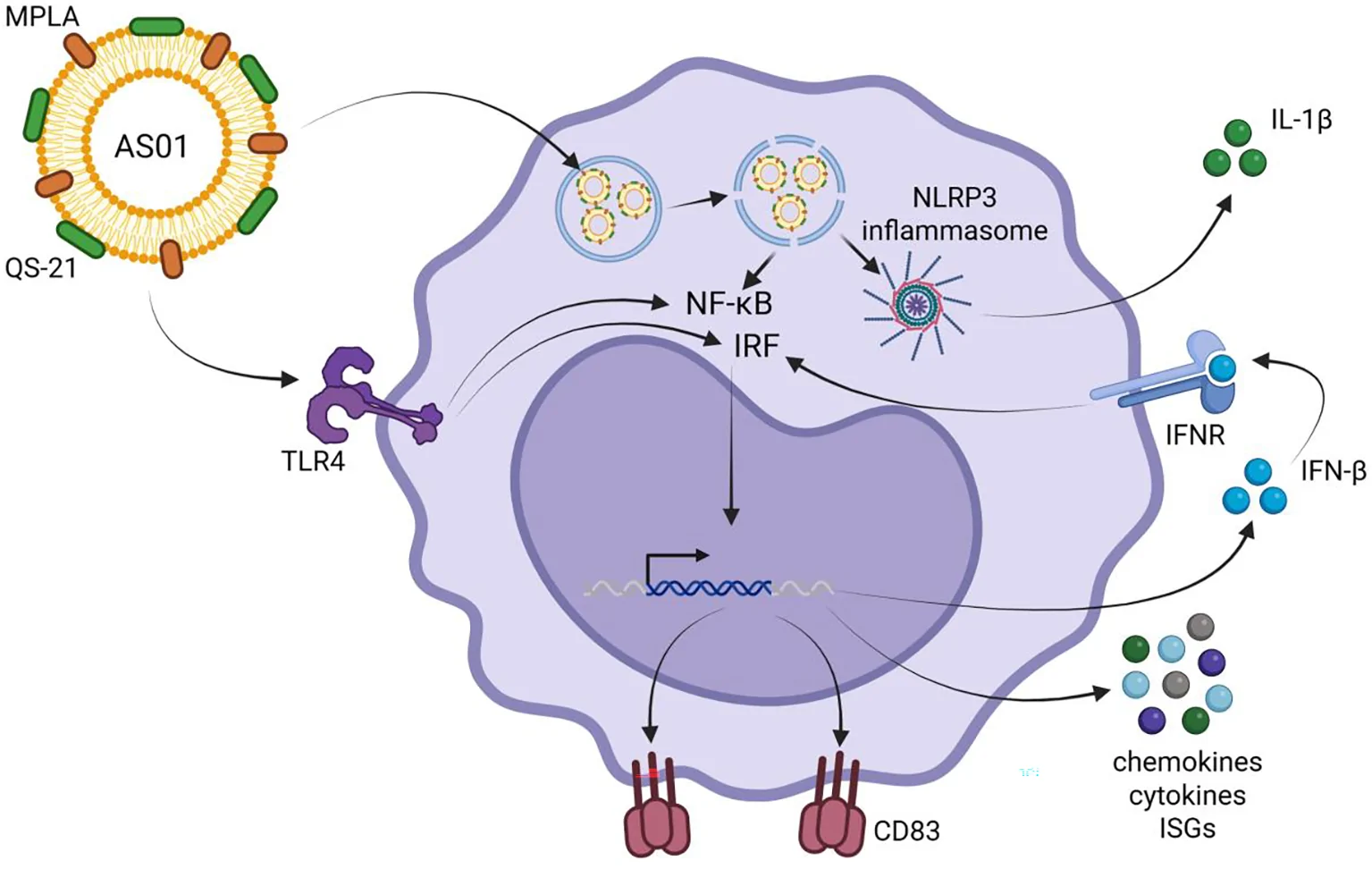
Summary of the effects of AS01 on THP-1 cells. AS01 contains the TLR4 ligand MPLA and the saponin QS-21 formulated in a liposome nanoparticle. Endocytosis of AS01 in lysosomes induces destabilization resulting in assembly of the NLRP3 inflammasome and secretion of IL-1β [ref. (19)]. Lysosomal destabilization and engagement of TLR4 synergize to activate the NF-κB signaling pathway. TLR4 activation also induces IRF activation resulting in secretion of the type I IFN, IFN-β. Autocrine signaling via the IFN-receptor (IFNR) stimulates IRF signaling. NF-κB and IRF induce transcription of genes encoding for chemokines, cytokines and interferon-stimulated genes (ISGs) as well as the membrane molecule CD83, a marker of dendritic cell maturation. Adapted from ref. (6). Created with Biorender.com.

## Data Availability

The raw data supporting the conclusions of this article will be made available by the authors, without undue reservation.
