# Supplementary material for: Dissecting the mechanism of action of the adjuvant system AS01 in human immune cells: synergy of QS-21 and monophosphoryl lipid A and role of type I interferons
Source: Front Immunol. 2026 Sep 24;17:1916491. doi: 10.3389/fimmu.2026.1916491 (PMC13646049; doi:10.3389/fimmu.2026.1916491)
Supplement: Supplementary file 1 [file DataSheet1.pdf]

Supplemental Information

Dissecting the mechanism of action of the adjuvant system AS01 in human immune cells: Synergy of QS-21 and monophosphoryl lipid A and role of type I interferons.

Michelle Poe[1,†], Nickolas Neokosmidis[1,†], Juan F. Hernandez[1], Alyssa Q. Stiving[2], Emory M. Payne[2], Shailaa Kummar[3], Jian Xiong[3], Angela Wagner[3], David A. Foley[2], Daniel J. DiStefano[2], Hualin Helen Li[2,*], Harm HogenEsch[1,4,*]

Supplementary Table 1. List of chemokines, cytokines and growth factors analyzed in the 96-plex assay (Eve Technologies, Human Cytokine/Chemokine 96-Plex Discovery Assay®)

APRIL	CCL17	CXCL6	G-CSF	IL-2	IL-15	IL-28A	sCD137
BAFF	CCL19	CXCL8	GM-CSF	IL-3	IL-16	IL-29	sCD40L
CCL1	CCL20	CXCL9	Granzyme A	IL-4	IL-17A	IL-31	SCF
CCL2	CCL21	CXCL10	Granzyme B	IL-5	IL-17E	IL-33	sFas
CCL3	CCL22	CXCL11	HMGB1	IL-6	IL-17F	IL-34	sFasL
CCL4	CCL23	CXCL12	IFN-α2	IL-7	IL-18	IL-35	TGF-α
CCL5	CCL24	CXCL13	IFN-β	IL-9	IL-20	LIF	TNF
CCL7	CCL26	CXCL16	IFN-γ	IL-10	IL-21	LT	TPO
CCL8	CCL27	CX3CL1	IFN-ω	IL-11	IL-22	M-CSF	TRAIL
CCL11	CCL28	EGF	IL-1RA	IL-12p40	IL-23	PDGF-AA	TSLP
CCL13	CXCL1	FGF-2	IL-1α	IL-12p70	IL-24	PDGF-AB/BB	VEGF-A
CCL15	CXCL5	FTL-3L	IL-1β	IL-13	IL-27	Perforin	XCL1

Supplementary Table 2. Fold change in chemokine and cytokine secretion by THP-1 stimulated with AS01 or LPS relative to medium control

Cytokine/chemokine	AS01 (mean ± SE)	LPS (mean ± SE)
CCL2	22.8 ± 1.6	2089.0 ± 254.16
CCL3	95.4 ± 2.01	8162.1 ± 1893.05
CCL4	995.1 ± 58.72	1053.9 ± 0
CCL8	171.1 ± 17.52	3561.4 ± 39.39
CCL24	3.0 ± 0.22	77.7 ± 19.21
CXCL1	12.1 ± 0.22	2199.0 ± 127.52
CXCL8	31.2 ± 0.31	532.8 ± 28.88
CXCL9	121.7 ± 9.16	922.9 ± 87.07
CXCL10	622.7 ± 55.94	1200.2 ± 172.76
CXCL12	2.1 ± 0.11	2.5 ± 0.14
IL-1β	2.5 ± 0.10	78.9 ± 6.37
IL-1RA	3.6 ± 0.23	1.9 ± 0.21
IL-18	2.9 ± 0.08	3.3 ± 0.41
IL-29	2.7 ± 0.10	2.0 ± 0.23
M-CSF	4.1 ± 0.24	2.9 ± 0.31
TNF	55.6 ± 4.48	1350.9 ± 181.46

Supplementary Table 3. Fold change in chemokine and cytokine secretion by human peripheral blood mononuclear cells (PBMCs) stimulated with AS01 or LPS relative to medium control

Cytokine/chemokine	AS01 (mean ± SE)	LPS (mean ± SE)
CCL1	44.7 ± 2.79	14.7 ± 1.63
CCL3	137.1 ± 10.87	208.9 ± 4.91
CCL4	23.7 ± 10.48	73.6 ± 34.00
CCL7	5.0 ± 0.17	-3.0 ± 0.17
CCL8	2.7 ± 0.14	-14.6 ± 0.51
CCL13	3.8 ± 0.03	-1.9 ± 0.05
CCL20	30.5 ± 0.36	200.8 ± 18.55
CCL22	1.6 ± 0.05	1.3 ± 0.08
CCL23	5.2 ± 0.33	1.2 ± 0.13
CCL28	2.0 ± 0.07	2.4 ± 0.91
CXCL1	27.3 ± 1.51	8.5 ± 0.47
CXCL6	2.9 ± 0.10	1.9 ± 0.14
CXCL8	3.3 ± 0.34	4.6 ± 0.24
CXCL11	2.5 ± 0.31	4.0 ± 0.24
IL-1α	150.6 ± 8.90	1359.7 ± 128.68
IL-1β	480.1 ± 16.29	9911.6 ± 1229.70
IL-1RA	10.6 ± 0.34	4.4 ± 0.38
IL-2	1.8 ± 0.13	2.3 ± 0.09
IL-6	182.6 ± 8.25	230.9 ± 12.24
IL-10	11.6 ± 1.31	18.8 ± 1.25
IL-12p40	29.2 ± 2.43	20.6 ± 0.31
IL-20	32.4 ± 1.41	21.4 ± 0.66
IL-23	9.1 ± 0.72	11.1 ± 0.66
G-CSF	203.0 ± 13.85	906.8 ± 37.32
GM-CSF	32.0 ± 1.80	45.5 ± 2.02
TNF	29.3 ± 1.17	166.3 ± 26.35

Supplementary Table 4. Fold change in chemokine and cytokine secretion by THP-1 cells stimulated with LP-QS-21, LP-PHAD or LP-QS-21/PHAD relative to empty LPs

Cytokine/chemokine	LP-QS-21	LP-PHAD	LP-QS-21/ PHAD
CCL2	17.9 ± 1.19	1.4 ± 0.09	55.2 ± 1.81
CCL3	81.1 ± 5.75	29.3 ± 3.84	337.6 ± 3.72
CCL4	150.4 ± 6.03	169.0 ± 19.64	15789 ± 0.0
CCL5	1.3 ± 0.10	1.8 ± 0.26	2.2 ± 0.07
CCL8	46.2 ± 4.64	2.2 ± 0.17	293 ± 6.62
CCL24	1.9 ± 0.07	1.2 ± 0.06	2.4 ± 0.13
CXCL1	2.6 ± 0.30	2.4 ± 0.26	38.7 ± 1.39
CXCL8	24.7 ± 0.65	13.5 ± 1.03	131.2 ± 7.14
CXCL9	110.1 ± 6.17	114.9 ± 18.17	332.8 ± 20.97
CXCL10	163.8 ± 30.52	41.1 ± 3.67	1219.6 ± 274.49
CXCL11	3.9 ± 0.51	2.1 ± 0.15	12.3 ± 0.33
CXCL12	2.2 ± 0.05	1.1 ± 0.04	2.4 ± 0.18
IL-1RA	7.9 ± 0.91	1.7 ± 0.22	11.9 ± 0.59
IL-12p40	0.7 ± 0.19	7.6 ± 0.20	7.0 ± 0.23
IL-29	10.3 ± 0.61	4.3 ± 0.55	14.3 ± 0.40
PDGF-AA	4.9 ± 0.22	1.8 ± 0.13	6.2 ± 0.17
TNF	34.8 ± 2.77	9.1 ± 0.65	70.9 ± 1.10
VEGF	1.5 ± 0.07	1.3 ± 0.11	1.8 ± 0.04

Supplementary Table 5. Primers used for qRT-PCR

Gene	PrimerBank ID	F primer	R primer	Amplicon size
IFIH1	27886567c3	TCACAAGTTGATGGTCCTCAAGT	CTGATGAGTTATTCTCCATGCCC	100
ISG15	193083170c1	CGCAGATCACCCAGAAGATCG	TTCGTCGCATTTGTCCACCA	152
MX1	222136618c3	GGTGGTCCCCAGTAATGTGG	CGTCAAGATTCCGATGGTCCT	97
OAS1	74229012c1	TGTCCAAGGTGGTAAAGGGTG	CCGGCGATTTAACTGATCCTG	122
IFNB1	50593016c1	ATGACCAACAAGTGTCTCCTCC	GGAATCCAAGCAAGTTGTAGCTC	88
IFNg	56786137c1	TCGGTAACTGACTTGAATGTCCA	TCGCTTCCCTGTTTTAGCTGC	93
IRF7	98985817c3	CCCACGCTATACCATCTACCT	GATGTCGTCATAGAGGCTGTTG	202
ACTB	4501885a1	CATGTACGTTGCTATCCAGGC	CTCCTTAATGTCACGCACGAT	250
GAPDH	378404907c2	ACAACTTTGGTATCGTGGAAGG	GCCATCACGCCACAGTTTC	101

Supplementary Table 6. Monoclonal antibodies used for flow cytometry

Marker	Fluorochrome	Clone	Source
HLA-DR	PE	L243	Biolegend
CD14	APC	M5E2	Biolegend
CD40	AlexaFluor 700	5C3	Biolegend
CD54	BV421	HA58	Biolegend
CD80	PE/Cyanine 7	2D10	Biolegend
CD83	FITC	HB15e	Biolegend
CD86	PE/Cyanine 5.5	IT2.2	Biolegend
